# Toleration games: compensatory growth by plants in response to enemy attack is an evolutionarily stable strategy

**DOI:** 10.1093/aobpla/ply035

**Published:** 2018-06-02

**Authors:** Gordon G McNickle, Wesley D Evans

**Affiliations:** 1 Department of Botany and Plant Pathology, Purdue University, West Lafayette, IN, USA; 2 Purdue Center for Plant Biology, Purdue University, West Lafayette, IN, USA

**Keywords:** Compensatory growth, evolutionary game theory, evolutionarily stable strategy, herbivory, plant allocation

## Abstract

Damage to plants from natural enemies is a ubiquitous feature of the natural world. Accordingly, plants have evolved a variety of strategies to deal with attack from enemies including the ability to simply tolerate attack. Tolerance often involves some form of compensatory response, such as the regrowth of tissues following damage. While ecological models of defence are common, there has been less effort to make predictions about the evolutionary stability of tolerance. Here, we present and experimentally test a game theoretic model of tolerance to herbivory. Plants in the model have a vector strategy which includes both root and shoot production, and herbivores in the model have a scalar strategy which is time spent foraging. The evolutionarily stable strategy (ESS) is the set of root growth, shoot growth and herbivore foraging which simultaneously maximizes all player’s fitness. Compensatory growth is not guaranteed, but it may emerge as an ESS if it maximizes plant fitness. We also experimentally tested the model predictions using wheat and simulated herbivory by clipping 0, 15, 30, 45 or 60 % of shoot production, and measured root, shoot and fruit production at senescence. The model predicted that compensatory growth was often an ESS when herbivores were either above- or below-ground. Plants in the experiment followed model predictions. Specifically, plants produced more tissues than expected based on damage, and for 15 % damage this allowed them to maintain equal fitness compared to undamaged plants. The model allows for above- and below-ground herbivory to be modelled, and predicts their impact on whole plant growth and reproduction. For example, we can predict the effects of shoot damage on root growth. When combined with other advances in predicting plant ecology with evolutionary game theory, we anticipate that this will be a valuable tool for generating further testable hypotheses.

## Teaser

Plants are regularly attacked by enemies such as herbivores and pathogens. Not surprisingly, plants have evolved ways of coping with attack. One way is to tolerate attack, and compensate for lost or damaged tissue with regrowth of additional tissue. In this paper, we develop an evolutionary game theoretic model that shows how tissue regrowth can be an evolutionarily stable strategy, and present results from an experiment that tests the model. Damaged plants in the experiment grew as expected from the model, which means the maths can be used as a new tool to understand plant–herbivore interactions.

## Introduction

Attack from natural enemies causes significant damage to plant tissues. Empirical estimates suggest that on average, plants in the field experience 18 % damage to above-ground tissues, and as much as 60 % of above-ground tissues can be removed (Cyr and Pace 1993; Milchunas and Lauenroth 1993). Indeed, damage done to plants by natural enemies can shape the structure and function of entire ecosystems (Pace *et al.* 1999; Ripple and Beschta 2012), potentially exerting control on the dominant vegetation type observed at the ecosystem level (Hairston *et al.* 1960; Whittaker 1975). This damage from enemies clearly has important fitness consequences for individual plants: when vegetative tissues are lost, resources (e.g. compounds synthesized from carbon, nitrogen and other minerals) that are contained in those tissues are also lost. Perhaps more importantly, damage to vegetative tissues from enemies also introduces an opportunity cost in terms of lost future foraging capacity: leaves that are removed or damaged cease to contribute future photosynthetic benefits to plants, and roots that are removed or damaged cease to contribute future nutrient and water uptake benefits. It is therefore not surprising that plants have evolved a variety of strategies in response to the selective pressures imposed by herbivores (Trumble *et al.* 1993; Rosenthal and Kotanen 1994).

The literature is not uniformly consistent with terminology, but logically, there are two basic classes of strategies that plants can employ as ways to resist damage from enemies: avoidance and tolerance (Rosenthal and Kotanen 1994; Krimmel and Pearse 2016). For sessile plants, avoidance primarily involves either defensive strategies (e.g. mechanical and chemical defence), or strategies that let plants escape the notice of enemies (Agrawal 2011; Johnson 2011). On the other hand, tolerance involves the degree to which plant fitness is altered by damage relative to an undamaged state, such that a perfectly tolerant plant would achieve equal fitness when damaged or undamaged (Strauss and Agrawal 1999). For example, many plants are able to exhibit compensatory responses that minimize the fitness costs of a damaged plant compared to an undamaged plant. These commonly include tissue regrowth strategies (Paige 1992; Paige 1999), but can also include compensatory changes in physiology (McNaughton 1983; Delaney 2008).

The evolution of plant strategies for defence is one of the best examples of a potential evolutionarily stable strategy (ESS) emerging from game theoretic frequency-dependent selection (McNickle and Dybzinski 2013). Game theoretic models of defence have examined a rich set of questions such as: the basic question of the evolution of defence versus no-defence (Oksanen 1990; Augner *et al.* 1991); unpalatability as a form of defence (Tuomi and Augner 1993); low nutrient content as a form of defence (Augner 1995); signalling of defences to herbivores (Augner 1994); Batesian mimicry of defence signals rather than actual defence (Augner and Bernays 1998); and even whether plants should be so toxic as to kill their herbivores (Tuomi *et al.* 1994). Different models make different assumptions and seek to capture different ecological phenomena. However, all models of defence somewhat intuitively show the same basic result: if the benefits of defence outweigh the costs, and herbivore feeding on defended and undefended mutants is dependent on the frequency of the two strategies, than defence should be expected to be the ESS. This means that it is important to recognize that the treatment of defence as an evolutionary game can also result the prediction that an absence of any of these defence-related strategies could be the ESS (Oksanen 1990), or that both strategies might coexist within one population (Augner *et al.* 1991; Tuomi and Augner 1993; Augner 1995). However, if plants exhibit some active form of tolerance such as regrowth of tissue following damage, then strictly speaking tolerance is not simply the absence of defence. There have been considerably less work ESS models of plant tolerance to herbivory. Here, our objective is to explore tolerance via compensatory growth as a potential ESS by developing a game theoretic model of plant enemy attack.

Previous approaches to modelling compensatory growth typically assume some plant-wide increase in compensatory growth following damage and show that there is potential for tolerance to outcompete other strategies in ecological terms (e.g. Järemo and Palmqvist 2001). However, such approaches do not make predictions about specific plant responses, such as root and shoot growth. Zhang *et al.* (2018) developed a full root-shoot model of plant compensation that allowed such predictions about root and shoot growth, and again were able to make predictions in ecological time. Both of these approaches create a model with a mathematical structure which is guaranteed to produce plant compensation, and clearly show that tolerance is potentially ecologically stable. However, by mathematically guaranteeing that compensation occurs, neither approach can explain how tolerance might evolve, or how it might be evolutionarily stable.

There have been several recent models that explore plant growth and allocation to leaves, stems, roots and reproductive tissues as an evolutionary game in response to plant–plant competition, and all of them have been shown to accurately predict global plant productivity across biomes (Dybzinski *et al.* 2011; McNickle *et al.* 2016; Falster *et al.* 2017). Although previous models did not include herbivory, they models provide a promising step forward in modelling tolerance to enemy attack as an ESS. In these models, the production of plant tissue itself is the game theoretic strategy, and the models predict the amount of growth that maximizes competitive ability among plants as an evolutionary game. For example, allocation to height can be beneficial when competition is above-ground (Givnish 1982; Falster and Westoby 2003), while allocation to roots can be beneficial when competition is below-ground (Schwinning and Weiner 1998; Gersani *et al.* 2001), and plants must make trade-offs in above- and below-ground growth (Reynolds and Pacala 1993; McNickle *et al.* 2016). Even though existing models predict production in many ecosystems (McNickle *et al.* 2016), developing an herbivory ‘module’ for game theoretic models of plant growth would be invaluable, since grassland ecosystems would be shrub or woodland in the absence of herbivory (Whittaker 1975).

To develop a game theoretic model of tolerance, we explore the consequences of introducing an herbivore into the game theoretic model of plant production developed by McNickle *et al.* (2016) to create a novel herbivory game. In the model, the plant strategy is a vector strategy of root and shoot production and the ESS is the tissue production that maximizes fitness in the presence of damage from an herbivore. Compensatory growth is not guaranteed in this model, but has the opportunity to arise as an ESS if it maximizes fitness. Because we model root and shoot growth together, this allows us to explore compensatory growth in roots, shoots and reproductive output simultaneously. Furthermore, plants in this model dynamically respond to resource availability via tissue growth strategies, and thus we are able to explore how compensatory growth is expected to interact with resource gradients.

First, using the model, we analyse ESS plant growth responses to fixed amounts of damage along a soil nitrogen gradient. The goal here is to develop predicted patterns in reproductive output, shoot and root growth that can subsequently be used as testable hypotheses. Second, using a greenhouse experiment, we test a subset of these model hypotheses along a gradient of simulated herbivory test whether the plants exhibited compensatory growth as predicted by the model. Interestingly, in all analyses, compensatory growth emerges as an ESS response to herbivory, which is constrained by resource availability, and has different effects when herbivores are above- or below-ground.

## Methods

### Model description

The model is a general foraging game among fixed numbers of individual plants and fixed numbers of individual enemies who are assumed only to interact with each other. The model assumes optimal foraging behaviour for both herbivores and plants. By optimal foraging we mean that plants are assumed to produce shoot material until the marginal benefits of carbon uptake balance the marginal costs of tissue production (equation 3, Table 1), and to produce roots until the marginal benefits of nutrient uptake balance the marginal costs of tissue production (equation 4, Table 1) (Hutchings and de Kroon 1994; Gleeson and Fry 1997). Plants are interesting in this foraging context because they have two spatially separated inputs (above- and below-ground) which must be balanced to optimize fitness defined as lifetime reproductive output. The herbivore also is assumed to forage optimally, and consume plant tissues as a resource. Here optimal foraging means that the herbivores are assumed to expend effort until the marginal benefits of plant tissue consumption balance the marginal costs of foraging effort (Charnov 1976; Brown 1988). Even though we refer to the plant enemy as an herbivore throughout, the mathematics are such that the enemy could be any type of organism that causes damage to plant tissues, including parasites and pathogens.

The herbivory game emerges because in the model herbivores adjust their foraging effort according to the tissue production of plants (i.e. food availability), and simultaneously plants adjust their leaf and root production (i.e. plant foraging effort) according to resources available in the environment, and how damage by the herbivore limits their access to these resources. The ESS is the plant and herbivore strategy that simultaneously maximizes both players’ fitness.

In this paper, we only analyse one plant attacked by one herbivore, and the solutions as well as the experiments described below reflect this choice. While it would be interesting to examine more complex scenarios with competing plants, this introduces the potential for competitive games to arise between plants (McNickle and Brown 2012; McNickle *et al.* 2016) that would complicate the interpretation of plant responses to herbivory. Similarly, the inclusion of multiple herbivores, potentially attacking plants above- and below-ground is more realistic and interesting, but it sets up a potential indirect game between herbivores mediated through the plant as they compete for plant tissues. This too would complicate interpretation. Thus, in this first description of the model, we analyse the simplest possible herbivory scenario to highlight and explore plant growth responses.

### Plant game

The plant model is a modified version of the model described in McNickle *et al.* (2016). The only difference is that McNickle *et al.* (2016) examined leaf, stem and root growth strategies, and here we collapse leaf and stem into a single shoot growth strategy (i.e. shoot biomass = leaf biomass + stem biomass). This is necessary because it is well known that without plant–plant competition there is no ESS solution for stem production (Givnish 1982; Falster and Westoby 2003).

Plant reproductive output (*G*, equation 1, Table 1) is a weighted product of net carbon harvest (*π*_*c*_) and net nitrogen harvests (*π*_*N*_). Net carbon (C) and nitrogen (N) harvest is weighted by the C:N ratio where: *α* is the fraction of plant seeds composed of carbon; *β* is the fraction of plant seeds composed of nitrogen, and it is assumed that *α* + *β* = 1 to ensure constant returns to scale as the plant grows. Even though we only include C and N in the model, it is worth noting that McNickle *et al.* (2016) showed how to mathematically include uptake of as many mineral nutrients as desired (e.g. nitrogen, phosphorus, potassium, …, boron, etc.) in this model, but also showed that the simplest model which included just carbon and nitrogen was adequate to predict global patterns in plant net primary productivity. The net harvest of carbon or nitrogen in the model is given by the resources harvested minus the resources required to construct and maintain roots and shoots (equations 2 and 3). For C or N, harvest increases with leaf or root production but with diminishing returns and saturates at some maximum harvest, while costs are assumed to linearly increase with tissue production.

**Table 1.** Fitness equations for the plant–herbivore game. Plant growth without herbivory is found by solving equation (1) with *u*_*h*_ = 0.

Description	Equation	Number
Plant fitness	$G(u_{r},u_{s},u_{h})=E\pi_{C}{}^{\alpha }\pi_{N}{}^{\beta }$	1
Plant harvest of C	$\begin{array}{cc} \pi_{C}(u_{r},\;u_{s},\;u_{h})=C\left(1-e^{-(V_{c}u_{s})}\right)-c_{rc}u_{r}-c_{sc}u_{s}-\alpha u_{i}\left(1-e^{-(au_{h})}\right),\; & where\;\{\begin{array}{c} \begin{array}{c} u_{i}=u_{s}\;for\;a\;shoot\;herbivore\\ u_{i}=u_{r}\;for\;a\;root\;herbivore\; \end{array}\\ u_{h}=0\;for\;no\;herbivore \end{array} \end{array}$	2
Plant harvest of N	$\begin{array}{cc} \pi_{N}(u_{r},\;u_{s},u_{h})=N\left(1-e^{-(V_{N}u_{r})}\right)-c_{rn}u_{r}-c_{sn}u_{s}-\beta u_{i}\left(1-e^{-(au_{h})}\right),\; & where\;\{\begin{array}{c} \begin{array}{c} u_{i}=u_{s}\;for\;a\;shoot\;herbivore\\ u_{i}=u_{r}\;for\;a\;root\;herbivore\; \end{array}\\ u_{h}=0\;for\;no\;herbivore \end{array} \end{array}$	3
Enemy fitness	$\begin{array}{cc} G(u_{h},\;u_{i})=u_{i}(1-e^{-au_{h}})-c_{h}u_{h}, & where\;\{\begin{array}{c} u_{i}=u_{s}\;for\;a\;shoot\;herbivore\\ u_{i}=u_{r}\;for\;a\;root\;herbivore\; \end{array} \end{array}\;$	4

### Herbivore game

For the herbivore, we assume the same basic functions and model structure where herbivore fitness is defined as plant tissue harvest minus costs (equation 4). This assumes that the herbivore comes to some equilibrium with the plant, which can include completely killing the plant. The resources consumed by the herbivore must obviously be removed from attacked plants. For an attacked plant, the cost is the amount of carbon contained in the consumed tissues, which is deducted from the net carbon gain (equation 2), and also the nitrogen contained in the consumed tissues, which is deducted from the net nitrogen gain (equation 3). Since *α* and *β* describe the fractional composition of plant tissues in terms of C and N, the amount of tissue consumed by the herbivore, $u_{i}(1-e^{-au_{h}})$ is multiplied by the C or N content of that tissue, and deducted from the plant’s net resource gain. In this way, herbivory removes both C and N from the plant regardless of whether the damage is above- or below-ground.

To model an undamaged plant in the absence of an herbivore, setting *u*_*h*_ = 0 would return the expected plant growth in the absence of herbivory.

### Evolutionarily stable strategies

From Table 1, the ESS production of roots and shoots (*u*_r_*, *u*_s_*) as well as the ESS foraging effort of the herbivore (*u*_h_*) occurs when the first partial derivative of each fitness function with respect to all strategies is equal to zero (Brown *et al.* 2007),

$$\begin{array}{cc} \begin{array}{l} \frac{\partial G(u_{r},u_{s},u_{h})}{\partial u_{r}}=\frac{\partial G(u_{r},u_{s},u_{h})}{\partial u_{s}}=\frac{\partial G(u_{i},u_{h})}{\partial u_{h}}=0,\\ where\{\begin{array}{c} u_{i}=u_{s}\;for\;a\;shoot\;herbivore\\ u_{i}=u_{r}\;for\;a\;root\;herbivore\\ u_{i}=0\;for\;no\;herbivore \end{array} \end{array} & \end{array}$$ (5)

and,

$$\begin{array}{l} \begin{array}{cccc} \frac{\partial^{2}G(u_{r},u_{s},u_{h})}{\partial u_{r}{}^{2}}<0,\; & \frac{\partial^{2}G(u_{r},u_{s},u_{h})}{\partial u_{s}{}^{2}}<0, & \frac{\partial^{2}G(u_{i},u_{h})}{\partial u_{h}{}^{2}}<0, & \end{array}\;\\ where\;\{\begin{array}{c} \begin{array}{c} u_{i}=u_{s}\;for\;a\;shoot\;herbivore\\ u_{i}=u_{r}\;for\;a\;root\;herbivore\; \end{array}\\ u_{i}=0\;for\;no\;herbivore \end{array} \end{array}$$ (6)

For the herbivore we can find an analytical solution that satisfies conditions (5) and (6), and the ESS for an herbivore is given by:

$$\begin{array}{cc} \frac{\partial G}{\partial u_{h}}=\frac{\partial H}{\partial u_{h}}-c_{h}=au_{i}{}^{*}e^{-au_{h}{}^{*}}-c_{h}=0,\; & where\;\{\begin{array}{c} u_{i}=u_{s}\;for\;a\;shoot\;herbivore\\ u_{i}=u_{r}\;for\;a\;root\;herbivore\; \end{array} \end{array}$$ (7)

From equation (7) if the plant growth strategy (*u*_i_*) is known, then,

$$\begin{array}{cc} u_{h}{}^{*}=\frac{1}{a}\text{ln}\left(\frac{au_{i}{}^{*}}{c_{h}}\right),\; & where\;\{\begin{array}{c} u_{i}=u_{s}\;for\;a\;shoot\;herbivore\\ u_{i}=u_{r}\;for\;a\;root\;herbivore\; \end{array} \end{array}$$ (8)

It should be clear from this analytical solution how high metabolic costs for the herbivore (*c*_*h*_) will reduce the foraging effort of the herbivore, and that low search efficiency (*a*_*h*_) can sometimes increase or sometimes decrease the foraging effort of foragers. Intuitively, increased plant growth also increases herbivore foraging effort. These parameters also interact.

For the plant there is no analytical solution that satisfies conditions (5) and (6). Indeed, the plant ESS is considerably more complicated because the plant uses a vector strategy that involves both root and shoot growth, and the fitness function is highly non-linear. From equation (1), the strategy of the plant satisfies:

$$\begin{array}{l} \frac{\partial G}{\partial u_{r}}=\alpha \pi_{c}{}^{\alpha -1}\pi_{n}{}^{\beta }\frac{\partial \pi_{c}}{\partial u_{r}}+\pi_{c}{}^{\alpha }\beta \pi_{N}{}^{\beta -1}\frac{\partial \pi_{N}}{\partial u_{r}}=0,\\ and\\ \frac{\partial G}{\partial u_{s}}={-}_{c}{{}^{--1}}_{n}{}^{†}\frac{\partial_{c}}{\partial u_{s}}{+}_{c}{}^{-}{†}_{N}{}^{†-1}\frac{\partial_{N}}{\partial u_{s}}=0 \end{array}$$ (9)

The partials required to fully expand equation (9) are given in Table 2 and must be found numerically. Because the herbivore ESS can always be found using an analytical solution, we primarily focus on plant responses for the remainder of the paper. Once the plant response $u_{i}{}^{*}$ is known, the herbivore strategy can always be found with equation (8).

**Table 2.** Partials required to expand and solve equation (9) for either an above- or below-ground herbivore. Setting *u*_*h*_ = 0, gives the solution for the growth strategy of an attacked plant.

Derivative with respect to	Carbon	Nitrogen
Roots	$\frac{\partial \pi_{C}}{\partial u_{r}}=-c_{cr}$	$\frac{\partial \pi_{N}}{\partial u_{r}}=V_{N}N(e^{-V_{N}u_{r}})-c_{nr}-\beta (1-e^{-au_{h}})$
Shoots	$\frac{\partial \pi_{C}}{\partial u_{s}}=V_{C}C(e^{-V_{c}u_{s}})-c_{cs}-\alpha (1-e^{-au_{h}})$	$\frac{\partial \pi_{N}}{\partial u_{s}}=-c_{ns}$

### Parameterization

McNickle *et al.* (2016) examined a wide distribution of parameters for plant physiology and growth based on observations in the literature, and validated these against two independent global data sets of plant net primary productivity (NPP). Therefore, we use their parameterization unless otherwise stated. These values are given in Table 3.

### Numerical simulations

Numerical solutions were performed using the multiroot() function in the rootSolve library (Soetaert 2009; Soetaert and Herman 2009) in the R Project for Statistical Computing (v. 2.13.0, R-Development-Core-Team 2009). Plant responses to damage were examined by holding the herbivore strategy constant and numerically finding ESS solutions to the plant growth strategy. This was done across a gradient of 50 different nitrogen availabilities. In this analysis, since damage is fixed, there are also no herbivore parameter effects (Table 3). As we describe below, this analysis was also chosen to reflect the treatments in a planned experiment.

**Table 3.** Parameter and variable descriptions and example units. Because this is a purely modelling exercise, the units shown are just examples, and in empirical applications of the model can be calibrated to different units as required by the biology of the system. Unless otherwise stated in the text, these values are the default values used in all analyses based on McNickle *et al.* (2016).

Plant parameters	Example units	Values	Description
*α*	NA	0.95	The ideal stoichiometric C:N ratio where *β* = 1 − *α* and the C:N ratio is *α*/*β*.
*V*_*n*_	gu_r_^−1^	1	The encounter rate between nitrogen uptake apparatus and molecules of inorganic nitrogen, which can be calculated from Michaelis–Menten uptake kinetics as, $\frac{V_{max}}{K_{m}}$ (McNickle and Brown 2014b).
*V*_*c*_	gu_s_^−1^	1	The encounter rate between photosynthetic apparatus and molecules of CO_2_, which can be calculated as the inverse of the maximum photosynthetic rate $\frac{V_{max}}{K_{c}}$.
*c*_*cs*_	gC (gu_s_)^−1^	3	The fractional amount of carbon required to construct a unit of shoot biomass. This includes total respiration costs and so might be greater than 1× standing biomass.
*c*_*cr*_	gC (gu_s_)^−1^	1.2	The fractional amount of carbon required to construct a unit of root biomass. This includes total respiration costs. This includes total respiration costs and so might be greater than 1× standing biomass.
*c*_*ns*_	gN (gu_r_)^−1^	0.03	The amount of nitrogen required to construct a unit of shoot biomass.
*c*_*nr*_	gN (gu_r_)^−1^	0.01	The amount of nitrogen required to construct a unit of root biomass.
*E*	gN^−1^gC^−1^	0.1	A conversion factor converting the product of resource uptake to reproductive output.
Herbivore parameters			
*a*	days^−1^	0.7	Encounter rate between herbivore and plant tissue. This can be thought of as search efficiency.
*c*_*h*_	(gu_i_) days^−1^	1	The mass of plant tissue (*u*_*i*_) required to fuel each time unit spent foraging. The variable *u*_*i*_ will be *u*_*s*_ for above-ground herbivores, and *u*_*r*_ for below-ground herbivores.
Environmental variables			
*C*	gC	5000	The maximum amount of carbon available to be acquired during the growing season.
*N*	gN	5–100	The maximum amount of nitrogen available to be acquired during the growing season.

Second, herbivore damage was solved dynamically with plant growth using the full set of model equations (Table 1). This was also done along the same gradient of nitrogen availability, for a shoot herbivore and a root herbivore separately.

### Metric of compensation

In all model and experimental analyses, we quantify compensation by calculating the log ratio of a production by a damaged and undamaged plant. Compensation as a log response ratio (lnRR) is a continuous metric of tolerance which defines the amount of regrowth following attack, and was calculated as,

$$lnRR=ln\left(\frac{u_{i,\;damaged}}{u_{i,\;undamaged}}\right)$$ (10)

From equation (10), positive lnRR values indicate over-compensation, negative lnRR values indicate under compensation and lnRR = 0 indicates perfect compensation. lnRR is calculated for reproductive output, shoots and roots in all scenarios.

Since the herbivore consumption is known in the model, proportion damage, *D*, can also be calculated, and is given by:

$$D=\frac{u_{i}{}^{*}(1-e^{-au_{h}{}^{*}})}{u_{i}{}^{*}}$$ (11)

where $u_{i}{}^{*}$ is replaced by $u_{s}{}^{*}$ for shoot herbivory or, $u_{r}{}^{*}$ for root herbivory.

### Experimental test of the model

We also performed an empirical test of a subset of predictions of the model. We grew wheat (*Triticum* sp., var. 9774-N2) in the greenhouse from September to December 2016. The wheat variety was chosen because of its low-tillering growth form and rapid 90-day life cycle (G. G. McNickle, pers. obs.). A grass was intentionally chosen because the simple root-shoot model structure did not include stems, and the growth form of grasses means that they do not have stems in the same way that a tree or other eudicots have stems. Grasses are also thought to possess many adaptations that make them tolerant to herbivory. The choice of a cereal crop was also intentional, since herbivory on crop plants has applied implications for food production and agroecology (Mitchell *et al.* 2016).

Plants were grown in 15-cm-diameter, 15-cm-tall pots at the Purdue University Lilly greenhouse (40.422704 N, −86.918685 W) for 90 days between September and December 2016. The soil was a 1:1 mixture of pre-sifted seedling mix (SunGro Propagation Mix, Sun Gro Horticulture Ltd, Agawam, MA, USA) and washed calcined clay gravel (Turface MVP, PROFILE Products LLC, Buffalo Grove, IL, USA). Soil was mixed with gravel to ensure the plants were nutrient limited, and to facilitate root harvest. Plants were watered every 3.5 days (Monday afternoon and Friday morning) during the 90-day growth period by pouring 250 mL of deionized water individually into each pot. Sunlight was supplemented by high-pressure sodium bulbs on a 16:8 h light:dark cycle, and the temperature was maintained at 22 °C.

Herbivory was simulated by clipping a percentage of each leaf by length once it was fully expanded using sharp scissors. Scissors are not a natural enemy of wheat, and it is well known that damage from biological enemies frequently elicits different responses than mechanical damage (Kessler *et al.* 2004; Bezemer and van Dam 2005). However, the model assumed only mechanical damage, and the goal was to experimentally test the model. Mechanical damage induces different gene expression than damage from herbivores, but mechanical damage does induce differences in gene expression in many plant species (Reymond *et al.* 2000). Thus, scissors were purposefully chosen to impose mechanical damage only as a direct test of our model. The damage treatments were 0, 15, 30, 45 and 60 % of the length of each fully expanded leaf clipped. Once a leaf was fully emerged, and the next leaf began developing, a ruler and scissors were used to impose the fixed percent damage treatment based on the actual length of the fully expanded leaf. Clippings of each plant were individually saved to be included as part of total production at the end of the experiment. Pots were arranged in a randomized block design to control for variation in the greenhouse benches, and there were 18 replicate blocks, for a total of 90 plants.

After 90 days, spikes were fully developed, and plant senescence had begun. At this time, spikes and shoots were clipped, dried at 60 °C and weighed. Soil and roots were stored until they could be washed by freezing at −20 °C. Thawed soil was washed on a 2-mm sieve to collect roots which were dried at 60 °C and weighed.

As with the model, we could calculate lnRR for the experimental plants by comparing each clipping treatment to the no-damage control from within a block. Additionally, because we clipped known percentages of the total shoot biomass, it was possible to use the undamaged controls in each block to create a floor for null expected growth in the absence of compensatory growth for the experiment just as we calculated in the numerical simulations. For example, if the shoot biomass of the undamaged plant is *x*, and the known proportion of the plant removed was *d*, then the expected lnRR in the absence of compensatory growth conveniently reduces to ln(1 − *d*), according to: $ln\frac{(1-d)x}{x}=ln(1-d)$.

### Statistical analyses

Since compensatory growth is defined as regrowth of vegetative tissues to maintain equal fitness of damaged plants compared to undamaged plants this means that a test of compensatory growth requires demonstration of both: (i) an ability to maintain fitness as a consequence of regrowth, and (ii) regrowth of the attacked vegetative tissue which we tested separately.

First, to test whether damaged plants were ever able to achieve similar fitness to undamaged plants (i.e. test for compensation), we fit a generalized linear mixed effects model with lnRR as the response variable and herbivory treatment as the independent variable and block as a random intercept in R. We then used *post hoc* planed comparisons to compare when damaged plants achieved equal fitness to undamaged plants.

Second, to test for regrowth of damaged shoot biomass, we compared observed and expected lnRR for different levels of shoot damage by fitting two separate linear mixed effects models, one for predicted lnRR versus the continuous damage treatment and one for observed lnRR versus the continuous damage treatment, both with block as a random effect. In this analysis, we treated damage as a continuous variable so we could estimate slopes and compare the slopes of the two models using a chi-squared test in R.

## Results

### Model predictions: fixed damage

**Figure 1.**
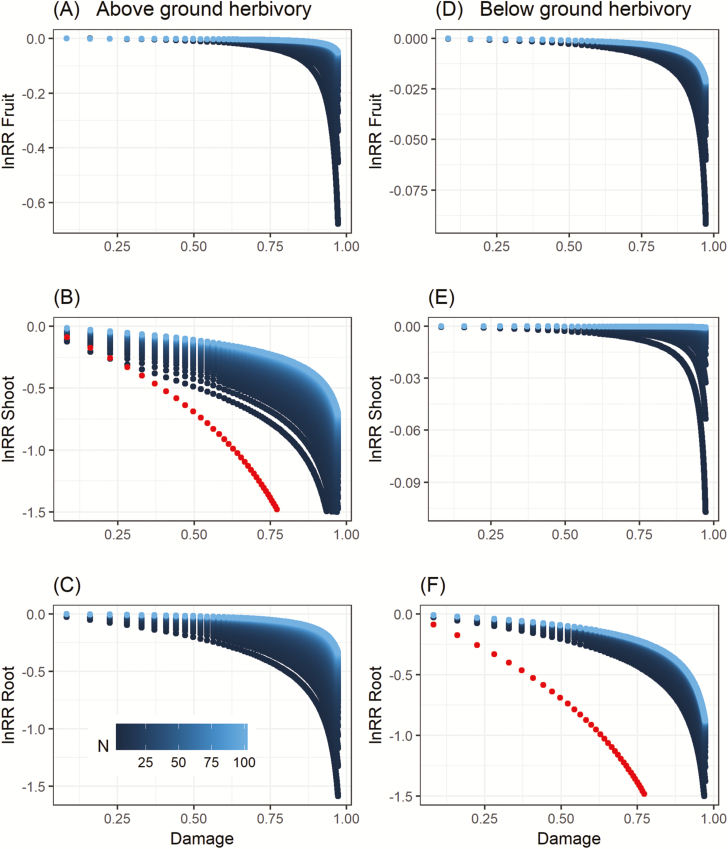
Model results for above-ground herbivory (left) and below-ground herbivory (right) with fifty different amounts of soil nitrogen (blue). Here, herbivory was fixed as shown on the x-axis and not allowed to vary with plant growth. Parameters were as in Table 3, except *N*_avail_ was varied as indicated in the figure legend. Compensatory growth is shown as log response ratios (lnRR) for (A, B) reproductive yield, (C, D) shoot biomass and (E, F) root biomass. For the damaged tissue, the red points show the null expectation if the plant did not respond with compensatory growth (C, F). Unfortunately, this null expectation cannot be calculated for undamaged tissues because of the complex non-linear trade-offs in root and shoot allocation (equation 1).

The model of plant growth predicted shoot biomass $u_{s}{}^{*}$ root biomass $u_{r}{}^{*}$ and the resulting lifetime reproductive output (our measure of fitness) achieved based on C and N uptake by the plant (*G*). Thus, we were able to look at compensatory growth responses in all three plant tissues. Not surprisingly, damaged plants are predicted to always be numerically smaller than undamaged plants (indicated by negative lnRR values, Fig. 1). However, in all modelling scenarios, the ESS growth strategy was one that sought compensatory growth (indicated by lnRR values close to zero, Fig. 1). In other words, damaged plants were never larger than undamaged plants, but were often nearly the same size. Plant damage is predicted to interact with N availability, such that plant ability to compensate for damage is intuitively higher when damage is low, but this ability is increased as N availability increases. We describe the results for an above- and below-ground herbivore separately below.

First, for an above-ground herbivore, the lnRR was close to zero for reproductive output at many levels of herbivory, indicating compensatory growth, and plant ability to tolerate herbivory increased with increasing N availability (Fig. 1A). Not surprisingly, when the herbivore damages the shoots, the most pronounced negative effects occur in shoot biomass production (Fig. 1B). Compensatory growth is still evident in that attacked plants are predicted to regrow shoot tissue following attack (blue points above red points), and it is this regrowth of shoot material that allows plants to maintain nearly constant reproductive output at all but the highest levels of damage, or lowest levels of N. Furthermore, the model also predicts that damaged plants are able to achieve nearly identical fitness to an undamaged plant at all but the highest levels of damage (Fig. 1A). In this integrated model of root and shoot growth, attack above-ground also leads to changes below-ground, with less overall root production in the presence of an above-ground herbivore (Fig. 1C). This is because in the integrated root-shoot model, increased compensatory shoot growth requires a trade-off that reduces root production, though this effect is quite small until damage approaches 100 %, or if nitrogen is highly limiting.

Second, the results for a below-ground herbivore are broadly similar to an above-ground herbivore. By dynamically adjusting their root and shoot growth following damage to the root system, damaged plants are predicted to be able to maintain nearly equal reproductive output compared to undamaged plants at all but the highest damage levels and lowest nitrogen levels (Fig. 1D). The shoot of a plant attacked below-ground shows a small response to the below-ground herbivore (Fig. 1E) which—as above—mainly reflects adjustments in shoot growth required to balance adjustments in root growth. Finally, as above, when the root system is damaged, the most obvious effects are in the root system (Fig. 1F).

### Model predictions: dynamic damage

**Figure 2.**
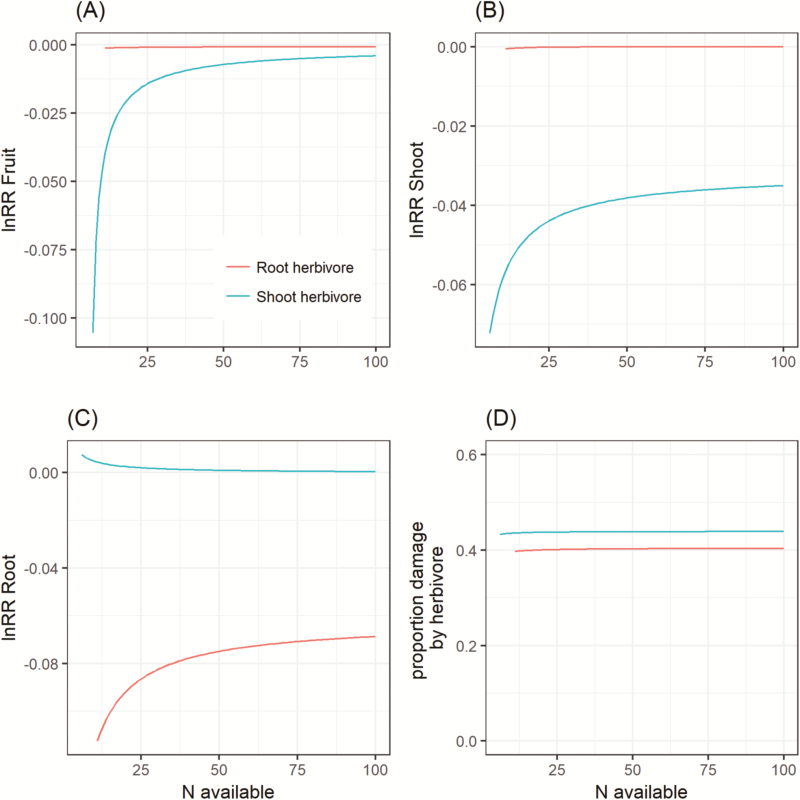
Model results for dynamic herbivory across a gradient of N availability. Both above-ground (blue) and below-ground (red) herbivory are shown for (A) plant fruit production; (B) plant shoot production; (C) plant root production; and (D) damaged caused by herbivore feeding. Parameters were as in Table 3, except *N*_avail_ was varied as indicated on the x-axis.

When herbivore damage was solved dynamically with plant growth, the predictions of the model were broadly similar to fixed damage described above. As above, shoot herbivores had a stronger effect on reproductive output then root herbivores, particularly at low N availability (Fig. 2A). Similarly, and not surprisingly, shoot herbivores had a larger negative effect on the shoot of a plant (Fig. 2B), while root herbivores had a larger effect on the root of the plant (Fig. 2C). Herbivores were predicted to have nearly constant foraging behaviour resulting in 43.8 % damage to the shoot and 40.2 % damage to the root averaged across nitrogen availability (Fig. 2D).

### Experimental results: fixed damage

The experimental results failed to reject the relevant hypotheses generated by model. Instead, the results of the fixed damaged experiment were broadly consistent with the predictions generated by the model at a single, low, N level.

**Figure 3.**
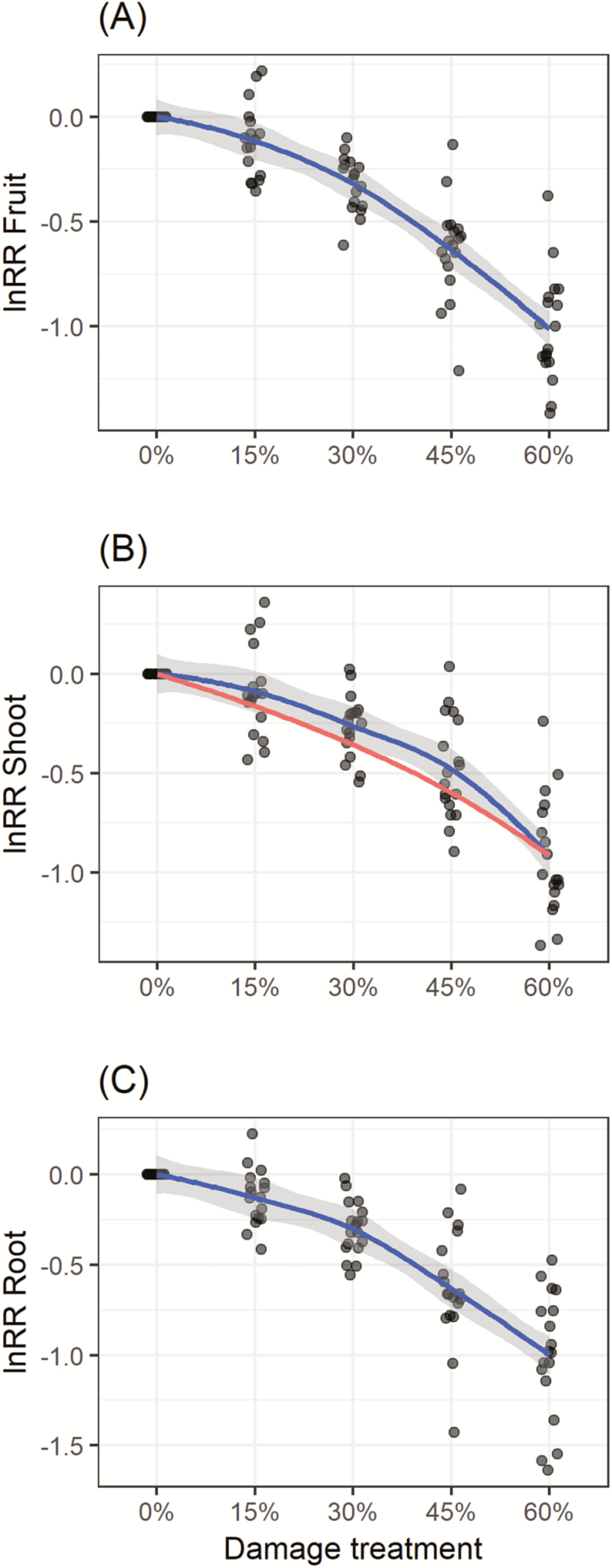
Experimental results testing a subset of predictions in Fig. 1. Growth of damaged plants relative to undamaged plants is shown as log response ratios (lnRR) for (A) reproductive yield, (B) total shoot biomass (standing + clipped) and (C) root biomass. As in the model results red lines in panel B show the null expectation if plants did not respond via compensatory growth but simply remained D% smaller than undamaged plants. Blue lines are loess fits with 95 % confidence intervals indicated by grey shading around the line. Individual points show the raw data with a jitter of ±0.1 damage.

First, we tested whether plants were ever able to maintain equal fitness when damaged relative to undamaged (e.g. model prediction, Fig. 1A). Overall, damage had a significant negative effect on plant reproductive output (*F*_4, 67.134_ = 116.76, *P* < 0.001, Fig. 3A). *Post hoc* comparisons revealed that plants were able to perfectly compensate for 15 % tissue removal (*t* = 2.126, *P* = 0.2214) such that they achieved equal fitness compared to undamaged plants (lnRR = 0, Fig. 3A). However, with 30 % (*t* = 5.964, *P* < 0.001), 45 % (*t* = 11.84, *P* < 0.001) and 60 % damage (*t* = 18.9, *P* < 0.001) *post hoc* comparisons revealed that plants achieved statistically lower fitness compared to undamaged plants indicating under-compensation. This is consistent with model predictions (Fig. 3A).

Second, we tested whether regrowth of the attacked vegetative tissue also occurred (e.g. model prediction, Fig. 1B). Overall, damage had a significant negative effect on shoot growth (*F*_1, 70.078_ = 152.13, *P* < 0.001). The slope of this observed lnRR versus damage relationship was significantly larger than predicted (χ^2^ = 263.8, *P* < 0.0001). Indeed, compensatory regrowth was evident from a shallower slope of observed damaged plants (Fig. 1B) (−1.078 ± 0.101 SE observed versus −1.511 ± 0.022 SE expected) and the intercepts were both close to zero (0.068 ± 0.037 SE observed versus 0.046 ± 0.008 SE expected) (Fig. 1B).

The model also predicts that root biomass should decline with increasing shoot damage (Fig. 1C), and indeed, as predicted there was an overall negative effect on root biomass (*F*_70.1, 1_ = 225.97, *P* < 0.001) even though roots were not directly damaged (Fig. 3C).

## Discussion

The model we have presented here provides one simple way to predict compensatory growth as a mechanism to tolerate herbivory, and the experimental results so far show that a subset of these predictions accurately reflect plant growth. Compensation by the plant was not forced into the model, but rather, emerged as the ESS solution to growth under herbivory when the model is made game theoretic. Indeed, when shoots were damaged, then the model predicted compensatory growth in the shoots (Fig. 1B), and when roots were damaged, then the model predicted compensatory growth in the roots (Fig. 1F). Both compensatory growth strategies theoretically led to tolerance in the sense that plants were predicted to be able to achieve similar fitness when damaged compared to undamaged (Fig. 1A and D). Because the model is an integrated model of root and shoot growth, we are also able to predict how roots are expected to respond to damage above-ground (Fig. 1C) and how shoots are expected to respond to damage below-ground (Fig. 1E). A subset of model predictions were tested for shoot damage only at a single low N availability, were supported by a greenhouse experiment that showed compensatory growth in damaged shoots (Fig. 3B) could allow similar fitness of damaged and undamaged plants (Fig. 3A) and declines in root biomass relative to undamaged plants (Fig. 3C). In general, all of these patterns have also been previously reported in other studies of herbivory or clipping. For example, experiments examining the effects of shoot herbivory typically find reductions in root biomass (Haag *et al.* 2004; Schadler *et al.* 2007). Similarly, experiments that manipulate root herbivores have shown reductions in shoot biomass as a result of damage to the root systems (Barber *et al.* 2015), though such experiments are considerably rarer.

The experiment presented here was designed as the simplest way to potentially falsify the model. However, the model makes a larger number of predictions then are tested by our single experiment. We suggest that the model should continue to be subjected to more severe empirical tests in the future. The most obvious next step in empirical scrutiny would be to include a factorial gradient of damage, and soil nitrogen availability and compare the growth of plants to the model predictions in Fig. 1. This ideally would include both root and shoot damage in a factorial design. It is interesting to note that the model predicts that plants might be more capable of tolerating attack below-ground compared to attack above-ground. For example, the model predicts that 50 % damage below-ground results in root systems of damaged plants that were between 88 and 99 % the size of undamaged plants, depending on N availability (Fig. 1F). By comparison, 50 % damage above-ground is predicted to result in shoots that are between 70 and 94 % the size of an undamaged plant, depending on N availability (Fig. 1B). This prediction also remains untested, and exploring the effects of damage to the root system would be another important test of the model. Finally, we suggest that testing the model predictions with a wider variety of plant species from a wider variety of functional groups would also shed light on limitations of the model.

In attempting to falsify the hypotheses in this model, we also suggest that more species should be examined. Compensatory growth is widely reported among many, but not all plant species (McNaughton 1983; Trumble *et al.* 1993; Järemo and Palmqvist 2001), and it is entirely possible that this variety of wheat was simply a fortuitous choice of study species. An excellent example of this is the recent application of game theoretic models to below-ground competition in plants, where simple models like the one presented here successfully predict the root growth behaviour of some species (Semchenko *et al.* 2010; Cahill and McNickle 2011; Padilla *et al.* 2013), while many other species seem to exhibit different responses (Dudley and File 2007; Smyeka and Herben 2017) and these different responses require different models to predict the root growth response (McNickle and Brown 2014a). Here, the use of lnRR meant that we only examined qualitative predictions of the difference between damaged and undamaged plants and so these qualitative predictions are much less sensitive to parameterization. However, the parameters in the model (Table 3) reflect physiological processes in plants that can be measured directly, or potentially obtained from the literature (e.g. McNickle *et al.* 2016), and might allow more precise quantitative predictions. For example, the parameter *α* defines the ideal stoichiometric C:N ratio, and therefore controls shoot and root allocation. Model plants parameterized with high values of *α* would therefore result in plants with lower root:shoot ratio compared to modelled plants with low values of *α*. Similarly, encounter rates between C (*V*_*c*_) and N (*V*_*N*_) are predictable from standard Michaelis–Menten uptake kinetics or photosynthesis models (McNickle and Brown 2014b), while cost parameters (*c*_*cs*_, *c*_*cr*_, *c*_*ns*_, *c*_*nr*_) define the amounts of C and N required to construct and maintain shoots and roots. Logically, plants with low ability to uptake C or N will have reduced capacity to regrow tissues and exhibit compensatory growth. Similarly, high cost tissues will be less able to exhibit compensatory growth compared to plants with low cost tissues. These patterns are part of the logic of the model and so were not evaluated in detail, but future applications to multiple genotypes or species could take the effects of different parameterizations into account to compare quantitative predictions.

The model also can make predictions about what to expect if an herbivore is allowed to go to equilibrium with the plant (Fig. 2). This is another hypothesis that remains untested. For example, small invertebrate herbivores can be enclosed around potted plants, permitting the measurement of both plant and herbivore behaviour. We view this as the most severe test to falsify the model we have presented. The dynamic herbivore equation does not apply to free-ranging herbivores such as ungulates who browse as they move, however, if the proportion of damage is known, then the dynamic plant response can still be predicted (e.g. Fig. 1).

It should be noted that the model equations can be changed to suit the needs of future researchers. For example, the model equations we used are the simplest possible, but they can easily be replaced with more mechanistic physiological models (e.g. Farquhar photosynthesis (Farquhar *et al.* 1980), or Michaelis–Mentent uptake kinetics (Michaelis and Menten 1913; Johnson and Goody 2011)) if this is desirable in future applications. There are a variety of existing plant growth models that take this more mechanistic approach which can be used for guidance (e.g. Dybzinski *et al.* 2011; Falster *et al.* 2017;  Zhang *et al.* 2018). However, these mechanistic models introduce an enormous amount of complexity into the plant growth model and this added complexity does not seem to improve model fit (see McNickle *et al.* 2016). Thus, we would caution against the loss of degrees of freedom unless the goal was to make predictions specifically about how plant physiology interacts with herbivory.

Another important consideration is that, we only modelled plant tolerance to herbivory. However, plants also have strategies for avoidance of herbivory, such as plant defence (Agrawal 2011; Johnson 2011). Importantly, avoidance and tolerance are not necessarily mutually exclusive (Rosenthal and Kotanen 1994; Krimmel and Pearse 2016). Plant defence strategies are a well-known example of an ESS (Augner *et al.* 1991; Augner 1994; Tuomi *et al.* 1994; Augner 1995; Augner and Bernays 1998). A future goal should be to integrate these models to examine trade-offs in avoidance and tolerance.

## Conclusions

In summary, our game theoretic model of plant growth under herbivory showed that compensatory growth is potentially an ESS. Whether damage was above- or below-ground plants were predicted to: (i) increase the production of the damaged tissue (Figs. 1B and F, and 2B and C), and (ii) this behaviour allowed the plants in the model to maintain almost equal fitness compared to undamaged plants, particularly when nutrients were abundant (Figs. 1A and D, and 2A). The model also makes predictions about how trade-offs in root-shoot allocation can lead to whole plant changes in growth (Figs. 1C and E, and 2B and C); (iii) finally, we can predict the dynamic behaviour of an herbivore–plant system, though this remains untested (Fig. 2D). We also experimentally tested a subset of model predictions along a gradient of simulated shoot damage at one level of N availability. From the experimental results, we conclude that wheat plants exhibited compensatory growth as an ESS strategy because they: (i) increased vegetative shoot growth following damage (Fig. 2B); (ii) this led to equal fitness of damaged and undamaged plants at low damage (Fig. 2A); and (iii) root growth was also negatively impacted by damage above-ground (Fig. 2B). These patterns of growth match the qualitative predictions of the model with a significant negative trend in the relationship between lnRR and damage for fruit production (Fig. 1A), shoot production (Fig. 1B) and root production as predicted by the model (Fig. 1A–C).

## Sources of Funding

This work was supported by the USDA National Institute of Food and Agriculture Hatch project 1010722.

## Contributions by the Authors

G.G.M. derived, solved and analysed the mathematical model. G.G.M. and W.D.E. designed the experiment. W.D.E. executed and harvested the experiment. G.G.M. and W.D.E. analysed empirical data. G.G.M. wrote the manuscript, and W.D.E. contributed to revisions.

## Conflict of Interest

None declared.
